# Incretin Response to Mixed Meal Challenge in Active Cushing’s Disease and after Pasireotide Therapy

**DOI:** 10.3390/ijms23095217

**Published:** 2022-05-06

**Authors:** Mattia Barbot, Alessandro Mondin, Daniela Regazzo, Valentina Guarnotta, Daniela Basso, Carla Giordano, Carla Scaroni, Filippo Ceccato

**Affiliations:** 1 Endocrinology Unit, Department of Medicine DIMED, University-Hospital of Padova, Via Ospedale Civile 105, 35128 Padova, Italy; alessandro.mondin.93@gmail.com (A.M.); daniela.regazzo@unipd.it (D.R.); carla.scaroni@unipd.it (C.S.); filippo.ceccato@unipd.it (F.C.); 2 Dipartimento di Promozione della Salute, Materno-Infantile, di Medicina Interna e Specialistica di Eccellenza “G. D’Alessandro”, UOC di Malattie Endocrine, del Ricambio e della Nutrizione, Università degli Studi di Palermo, Piazza delle Cliniche 2, 90127 Palermo, Italy; valentina.guarnotta@unipa.it (V.G.); carla.giordano@unipa.it (C.G.); 3 Laboratory Medicine Unit, Department of Medicine DIMED, University-Hospital of Padova, 35128 Padova, Italy; daniela.basso@unipd.it

**Keywords:** Cushing’s disease, mixed meal test tolerance test, diabetes mellitus, incretin, pasireotide

## Abstract

Cushing’s disease (CD) causes diabetes mellitus (DM) through different mechanisms in a significant proportion of patients. Glucose metabolism has rarely been assessed with appropriate testing in CD; we aimed to evaluate hormonal response to a mixed meal tolerance test (MMTT) in CD patients and analyzed the effect of pasireotide (PAS) on glucose homeostasis. To assess gastro-entero-pancreatic hormones response in diabetic (DM+) and non-diabetic (DM–) patients, 26 patients with CD underwent an MMTT. Ten patients were submitted to a second MMTT after two months of PAS 600 µg twice daily. The DM+ group had significantly higher BMI, waist circumference, glycemia, HbA1c, ACTH levels and insulin resistance indexes than DM− (*p* < 0.05). Moreover, DM+ patients exhibited increased C-peptide (*p* = 0.004) and glucose area under the curve (AUC) (*p* = 0.021) during MMTT, with a blunted insulinotropic peptide (GIP) response (*p* = 0.035). Glucagon levels were similar in both groups, showing a quick rise after meals. No difference in estimated insulin secretion and insulin:glucagon ratio was found. After two months, PAS induced an increase in both fasting glycemia and HbA1c compared to baseline (*p* < 0.05). However, this glucose trend after meal did not worsen despite the blunted insulin and C-peptide response to MMTT. After PAS treatment, patients exhibited reduced insulin secretion (*p* = 0.005) and resistance (*p* = 0.007) indexes. Conversely, glucagon did not change with a consequent impairment of insulin:glucagon ratio (*p* = 0.009). No significant differences were observed in incretins basal and meal-induced levels. Insulin resistance confirmed its pivotal role in glucocorticoid-induced DM. A blunted GIP response to MMTT in the DM+ group might suggest a potential inhibitory role of hypercortisolism on enteropancreatic axis. As expected, PAS reduced insulin secretion but also induced an improvement in insulin sensitivity as a result of cortisol reduction. No differences in incretin response to MMTT were recorded during PAS therapy. The discrepancy between insulin and glucagon trends while on PAS may be an important pathophysiological mechanism in this iatrogenic DM; hence restoring insulin:glucagon ratio by either enhancing insulin secretion or reducing glucagon tone can be a potential therapeutic target.

## 1. Introduction

Cushing’s disease (CD) is the most common cause of endogenous cortisol excess. CD is burdened by cardiovascular and metabolic complications, of which diabetes mellitus (DM) is one of the most frequent. The overall prevalence of glucose metabolism impairments reaches nearly 70% of cases, including both overt DM and prediabetes [1]. Since glycemic impairment due to cortisol excess is associated especially with impaired postprandial glucose metabolism, the effective extent of DM in CD is probably underdiagnosed as in most cases only fasting glycemia is available and adequate provocative testing is not routinely performed. Among the mechanisms behind glucocorticoid (GC)-induced DM, insulin resistance is probably the most relevant, even though several other factors contribute to impair glucose homeostasis, such as β-cell dysfunction, altered glycogenolysis and hepatic glucose release [2,3].

Despite DM representing a recognized independent risk factor for increased mortality, it has only been marginally studied in CD patients. Both mechanistic and therapeutic studies on endogenous hypercortisolism are scant and specific treatment recommendations for these patients derive from large studies on patients with type 2 DM [4].

Furthermore, to our knowledge, only very few studies have described the effect of GCs on the secretion of incretin hormones [5,6,7]. Incretin hormones are primarily produced by enteroendocrine cells of the gut and secreted into the blood stream after meal ingestion in order to control post-prandial glucose peak through their insulinotropic effect. Notably, nutrients other than glucose (e.g., protein, fat) have been shown to stimulate insulin secretion and incretin levels [8]. Thus, MMTT is preferable to oral glucose tolerance test (OGTT) to assess both the pancreatic function as well as the integrity of the entero-insular axis through incretin hormones release. Furthermore, it represents a more physiologically relevant challenge, mimicking daily life situations [9].

Although treatment indications for GC-induced DM are similar to that of type 2 DM, in CD the treatment of the underlying condition is the key point to control glucose impairment [10]. However, it should be recalled that different treatments for hypercortisolism may affect the outcome of DM, regardless of how well cortisol excess is controlled.

The first-line treatment for CD is transsphenoidal surgery (TSS); when effective it frequently results in a resolution of cortisol-related comorbidities, even though metabolic alterations may persist after remission in some cases [11]. Unfortunately, up to 50% of CD cases persist or recur after TSS and second-tier options, such as medical therapy, are required to control cortisol excess [12].

Medical therapy is not curative, but can at least revert most cortisol-related complications through the reduction of hormone levels. Unlike other available drugs, the somatostatin receptor multiligand pasireotide (PAS) causes a worsening of glucose metabolism in about three-quarter of cases [13]. PAS administration produces a rapid decrease in insulin secretion with a consequent increase in glucose levels, as we previously reported. The incretin system was also partially affected but to a less extent compared to that observed in healthy subjects [14,15].

However, there are no data available on the effect of PAS on the incretin system and its response to meal stimulus in everyday life of CD patients while on this chronic treatment.

In this study, we evaluated the impact of cortisol excess on glucose homeostasis through a standard MMTT to investigate the response of these hormones in patients with overt CD. In a subset of patients, we also evaluated the effect of two months of PAS therapy on gastro-entero-pancreatic hormonal response to MMTT in order to further comprehend the mechanisms leading to PAS-related DM and provide clues about the best treatment choice in this particular setting.

## 2. Material and Methods

Twenty-six patients (twenty females, six males; median age 45 years, range 27–79 years) with CD were prospectively enrolled for this study. CD diagnosis was established based on Endocrine Society Guidelines [16]. Among them, 15 cases had previous TSS resulting in persistent or relapsing disease, one underwent primary pituitary radiotherapy, whereas 10 were treatment-naïve patients. Patients previously treated with other cortisol-lowering medications (ketoconazole or metyrapone) observed a wash-out period of at least a week before testing.

All patients underwent an MMTT after an overnight fasting of at least 12 h. The meal consisted of: orange juice (125 mL), bread (80 g), raw ham (50 g) and Grana cheese (50 g), for a total of 565.5 kcal (30% of proteins, 55% of carbohydrates and 15% of fats). Patients were instructed to consume the meal rapidly (within 10 min). Blood samples for ACTH, cortisol, glucose, insulin, C-peptide, glucose-dependent insulinotropic peptide (GIP), glucagon-like peptide 1 (GLP-1) and glucagon measurements were collected right before the meal (0′) and at 15′, 30′, 60′, 90′, 120′, 150′ and 180′ after MMTT. 

At baseline 7/26 patients presented impaired glucose homeostasis (DM+ group), with 3 patients presenting overt DM and 4 presenting impaired fasting glucose (IFG) according to ADA guidelines [4]. Among patients with overt diabetes, two patients did not receive any pharmacological treatment while the other was on metformin 1500 mg/d.

After the initial testing, seven patients did not start PAS administration due to personal preference or clinician’s evaluation favoring different approaches. The remaining 19 patients started PAS administration between 2013 and 2019, with a median treatment period of 6 months (interquartile range (2–26) months). Among them, 10 accepted to undergo a second MMTT after two months of PAS treatment; only one of them was in the DM+ group.

Clinical evaluation including weight, height, body mass index (BMI), waist circumference and blood pressure measurements as well as biochemical testing were performed both at baseline and after 2 months of therapy. We also performed biochemical assays for fasting blood glucose, glycated hemoglobin (HbA1c) and lipid profile.

All subjects gave their informed consent for inclusion before they participated in the study. The study was conducted in accordance with the Declaration of Helsinki, and the protocol was approved by the Ethics Committee of Padova (project identification code: 4834/AO/20).

A Roche COBAS 8000 automated modular analyzer was deployed to measure triglycerides and total and high-density lipoprotein (HDL) cholesterol serum levels, while low density lipoprotein (LDL) cholesterol levels were estimated through Friedewald equation. Fasting glycemia was assessed with an enzymatic method (Yellow Springs Glucose 2300 STAT-Analytical ServiceSRL). HbA1c was assessed with an HPLC method coupled with indirect UV-spectrophotometric detection assay (Biorad D10, Biorad, Milan, Italy). Plasma ACTH and serum cortisol levels were measured by immunochemiluminescence (Immulite 2000, Siemens Healthcare, Erlangen, Germany), while liquid chromatography coupled with tandem mass spectrometry (LC-MS/MS) was performed for urinary free cortisol (UFC) and late-night salivary cortisol (LNSC). Salivary samples were drawn thorough specific devices (Salivette), instructing patients to avoid interfering factors such as smoke, tooth paste or licorice. Glucagon and incretin dosages were performed through ELISA immunoassays (GIP-EMD Millipore Corporation, Billerica, MA, USA; GLP1 and glucagon-Mercordia, Uppsala, Sweden). Plasma insulin and C-peptide levels were measured by chemiluminescent immunometric assay (Siemens Automated Immunolite 2000, Siemens Medical Solutions Diagnostic, Malvern, PA, USA).

We indirectly estimated basal insulin secretion through HOMA-B index (HOMA-B = ((360 × insulin)/(glycemia-63))%) and insulin sensitivity through homeostatic model of insulin resistance HOMA-IR index (HOMA-IR = (glycemia × insulinemia/22.5)). Insulin sensitivity was also estimated with the quantitative insulin sensitivity check index (QUICKI) using the following formula: QUICKI = 1/((log(fasting insulin) + log(fasting glucose)). The fasting insulin resistance index (FIRI), consisting of the product of plasma insulin and glucose, was obtained using the formula FIRI = (fasting glucose × fasting insulin)/25.

Patients’ demographic and clinical characteristics at baseline were reported as count and percentage in case of categorical variables or as median and interquartile ranges (IQR) for quantitative variables. Comparisons between groups were drawn with a Mann–Whitney sum rank test for independent variables, while a Wilcoxon signed-rank test was performed for dependent variables. A chi-square test was performed to confront categorical variables. Total areas under the curve (AUC) were computed for each parameter according to the trapezoidal formula. Net increase (Δ) above baseline was calculated as the difference between absolute peak and basal value. Net AUC (nAUC) is calculated by applying the trapezoid rule to both positive and negative variations; thus, it is calculated by subtracting the area below the fasting level from the above. The threshold for statistical significance was set at *p*-value < 0.05. Statistical analysis was performed using SPSS software for Windows, version 17.0.

## 3. Results

### 3.1. Baseline Metabolic Profile

**Figure 1:**
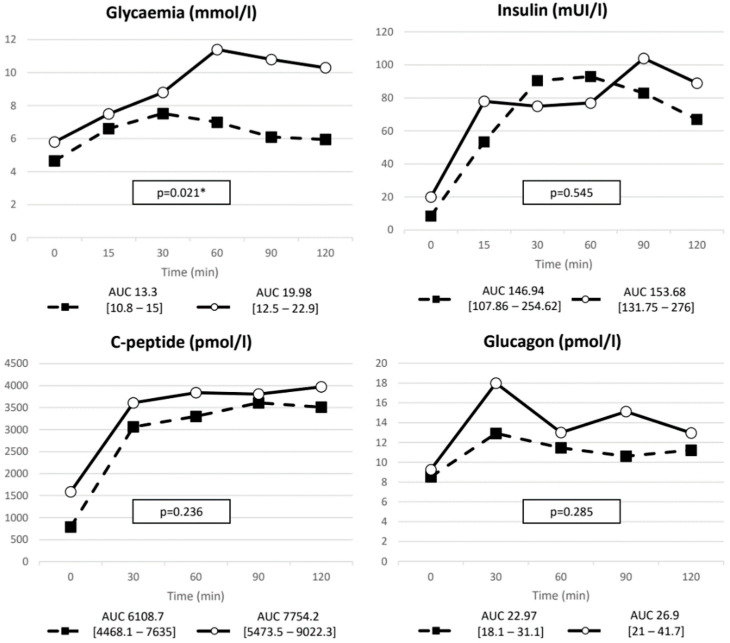
Glycemia, insulin, C-peptide and glucagon AUC after MMTT. Solid line, DM+ patients; dashed line, DM− patients. * *p* value < 0.05.

Baseline evaluation showed that DM+ patients displayed more features of metabolic syndrome, such as higher BMI and waist circumference compared to DM− patients, but comparable UFC levels (Table 1). This clinical phenotype was associated with higher C-peptide levels along with a tendency to have increased fasting GIP levels. The DM+ group also showed higher indexes of insulin resistance, while no significant difference was found in insulin secretion and in insulin:glucagon ratio. No differences were observed for the degree of hypercortisolism, whereas higher baseline ACTH values were recorded for DM+ patients (Table 1). No difference in macroadenomas distribution was observed between groups (DM+ 33.3% vs. DM− 45.5%, *p* = 0.926). Familiar history of diabetes was more frequently found in the DM+ group (*p* < 0.001). No differences in duration of disease were found between DM+ and DM− groups (*p* = 0.852). Despite baseline differences, insulin response did not exhibit a greater response to MMTT in the DM+ group. Regarding glycemia, the AUC was significantly higher in DM+ patients, whereas baseline and meal response did not differ (Figure 1 and Table 2). 

**Figure 2:**
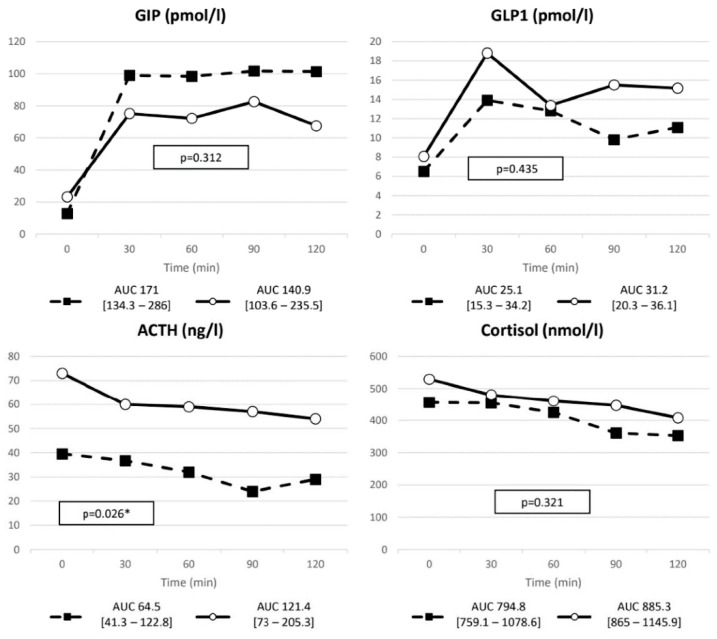
GIP, GLP-1, ACTH and cortisol AUC after MMTT. Solid line, DM+ patients; dashed line, DM− patients. * *p* value < 0.05.

In both DM+ and DM− groups, glucagon significantly rose after meal stimulation (baseline vs. peak DM+: 9.25 (9.15–14.72) vs. 18 (11–34.8) pmol/L, *p* = 0.018; baseline vs. peak DM−: 8.53 (3.86–12.75) vs. 12.92 (8.12–18.43) pmol/L, *p* = 0.001). No significant differences in fasting glucagon nor in meal-stimulated levels were found between the two groups (Figure 1). Conversely, GIP increase was lower in the DM+ group compared to the DM− (nAUC respectively 89.87 (55.36–134.35) vs. 148.71 (106.5–221), *p* = 0.035). No differences were observed for GLP-1 between the two groups (Figure 2).

### 3.2. Results PAS

After two months of PAS therapy, UFC was significantly reduced compared to baseline (Table 3). As a result of PAS action, fasting glycemia (4.8 (4.5–5.1) vs. 5.3 (5–7.6) mmol/L, *p* = 0.009) and HbA1c (35 (29.5–36.5) vs. 41 (36.75–63.25), *p* = 0.012) increased during this time frame. The two months of PAS treatment induced a reduction of HOMA-B and insulin resistance (HOMA-IR). This effect was obtained without any significant differences in BMI and waist circumference after therapy (Table 3).

**Figure 3:**
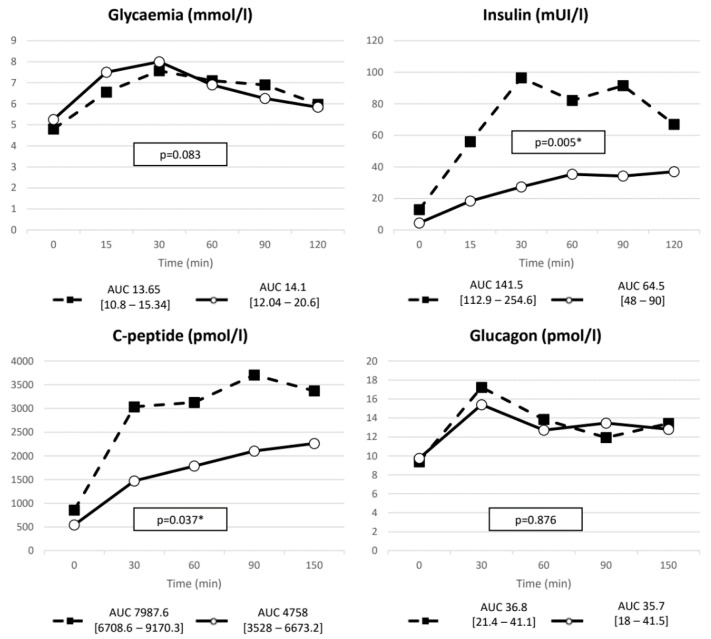
Glycemia, insulin, C-peptide and glucagon AUC after the meal. Dashed line represents pre-treatment values whereas solid line the post-treatment curve. * *p* value < 0.05.

**Figure 4:**
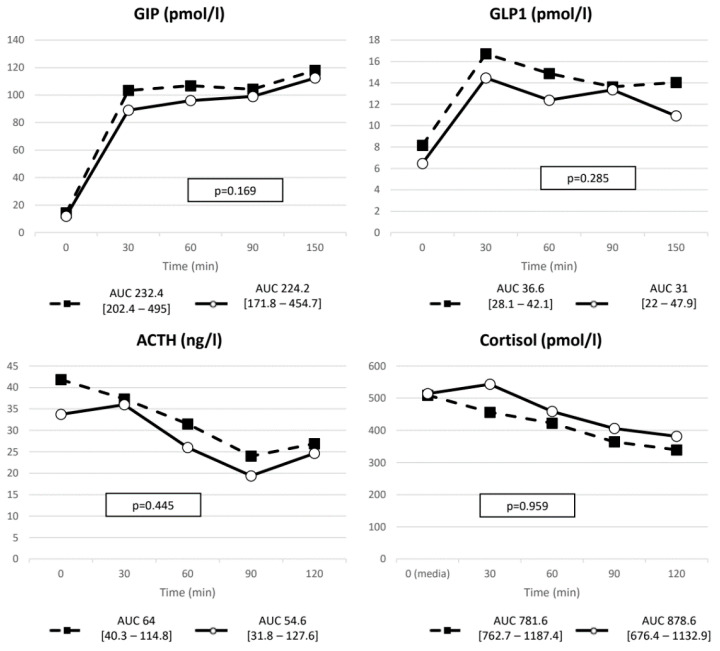
GIP, GLP-1, ACTH and cortisol AUC after the meal. Dashed line represents pre-treatment values whereas solid line the post-treatment curve.

Both insulin and C-peptide were reduced in fasting condition and exhibited a blunted response to MMTT in terms of peak to baseline and nAUC compared to pre-treatment levels (Table 3 and Table 4). Glucose curve after two months of PAS was instead similar to baseline evaluation, despite the aforementioned decrease in insulin secretion. Glucagon response remained basically unchanged after PAS therapy (Figure 3). As a consequence, a lower insulin:glucagon ratio was recorded after PAS treatment (Table 3). No differences were found in incretin basal and meal-induced levels. ACTH and cortisol curves after meal were not modified by two months of PAS therapy (Figure 4).

## 4. Discussion

This study for the first time systematically evaluated dynamic changes in β-cell function, insulin resistance and incretin hormones in diabetic and non-diabetic CD patients under a physiological stimulus such as mixed meals. Despite the common diabetogenic background represented by cortisol excess, DM+ patients exhibited higher insulin resistance (IR) but comparable fasting glycemia than DM− cases. This finding is in line with the increased IR in cortisol-induced DM and a common trait in type 2 DM, as well as the more frequent familiar history of impaired glucose metabolism. In our study no difference in insulin secretion (estimated through HOMA B index) was found, even though other authors also described a cortisol inhibitory effect on insulin secretion. Indeed, GC receptor is expressed in pancreatic β-cells [17] and cortisol excess seems to reduce insulin secretion through multiple intracellular signalling pathways including increased β-cell apoptosis [2]. This might depend on the population recruited, as 4/7 DM+ cases exhibited impaired fasting glucose, suggesting an early phase of glucose metabolism impairment, with preserved β-cell functionality. Thus, steroid-induced insulin resistance may lead to compensatory insulin oversecretion, that conceals the effects of cortisol excess on the β-cell function, as suggested by increased C-peptide levels in DM+ patients.

Despite a trend to higher baseline levels, we observed a blunted GIP response to MMTT in the DM + group, potentially related to the higher BMI in this group, as observed in type 2 diabetic patients after MTT. However, a potential indirect inhibitory effect of cortisol excess on insulin secretion, through the decreased past-prandial incretin stimulation cannot be ruled out [18]. Overall, the secretion of GIP was similar between groups as previously demonstrated in first-degree relatives of patients with type 2 DM experimentally treated with short term dexamethasone [6]. Likewise, GLP-1 was not reduced in DM+ patients; this is in line with available data on patients with recent DM onset, especially in impaired glucose tolerant cases [19]. Since incretin concentrations are almost normal in CD, cortisol excess might act mainly through the impairment of the incretin effect by reducing protein kinase A-mediated insulin release [5,20].

Both in DM+ and DM− groups, glucagon rose significantly after meal stimulation; this finding has already been described in patients with IFG [21] and a positive correlation between insulin resistance and hyperglucagonemia has been observed in subjects with impaired glucose tolerance [22]. The relative hyperglucagonemia in DM− patients can be related to cortisol excess per se, as previously found in healthy subjects when treated with exogenous GCs [19]. Therefore, elevated post-prandial glucagon levels might represent a potential underlying mechanism involved in post-prandial hyperglycemia and progression to DM in CD [19]. 

In our series there was no difference in cortisol levels, but higher ACTH levels in the DM+ population were found. This difference does not appear to be related to the population recruited, since the proportion of macroadenomas was similar in the two groups. ACTH elevation is usually only indirectly associated to diabetes as it causes cortisol excess, which is the major determinant of DM in CD. However, a direct effect of ACTH on glucose homeostasis cannot be excluded, especially considering that ACTH has been proved to induce insulin resistance in vitro [23]. High ACTH level was already reported in our previous study as a potential contributor to DM development during PAS therapy [15]. Further studies are required to obtain a deeper understanding of these mechanisms. Similarly, duration of hypercortisolism exposure did not differ between DM+ and DM− patients; still, it should be recalled that CD diagnosis can be delayed in some cases, thus hampering the real meaning of this result.

In cortisol-related DM, the key strategy is hormone normalization. When surgery proves ineffective, medical therapy is a valuable option to achieve disease control. As opposed to other drugs, PAS is burdened by a significant risk of iatrogenic DM despite its effectiveness in controlling UFC [12]. It has been reported that PAS-related hyperglycemia originates from impaired insulin secretion. PAS inhibits insulin secretion both directly, through somatostatin receptors (SSTRs) 1-3-5 that are strongly expressed in the pancreatic β-cell [24], and indirectly, by reducing post-prandial incretin secretion. These effects prompted by a consequent increase in glucose levels, were observed in healthy volunteers after PAS administration; no changes in insulin sensitivity was described [14].

In our population, after two months of PAS administration, we observed significant reduction in insulin and C-peptide baseline secretion, as well as an increase in fasting glycemia. Both estimated insulin secretion and resistance indexes decreased significantly while on therapy. While the effect on insulin secretion was expected, no previous studies reported an improvement in insulin resistance during PAS therapy [25]. Indeed, drug-induced cortisol reduction is probably responsible for the amelioration of insulin sensitivity; notably the effect was obtained irrespective of the improvement of clinical features, such as BMI and waist circumference, strengthening the pivotal contribution of cortisol to insulin resistance in CD. This finding may also explain why, even though meal-induced C-peptide and insulin responses were significantly impaired, there was no difference in glucose trend during MMTT.

A possible confounding factor is the introduction of metformin treatment in patients developing DM, but it was the case only in one patient. 

As opposed to previous data on healthy volunteers, no differences in baseline and meal-induced incretin levels were observed [14]. Still, it should be mentioned that this discrepancy may also be related to the different study protocol as the last dose of PAS was administered the night before testing and not in the morning of MMTT. In our study we confirmed an inhibitory effect of PAS therapy on insulin secretion without a significant decrease in meal-induced incretin production, suggesting a main role of direct inhibition of β-cell activity via SSTR-5. However, other mechanisms might explain this finding; indeed, a reduced intra-islet paracrine effect of GLP-1 cannot be ruled out whereas an increased interleukine-6 mediated GLP1 secretion in CD may disguise PAS inhibitory effect [26].

It has also been reported that a single PAS injection also inhibits α-cell activity, causing a decrease in glucagon secretion [15]. Vice versa, in the present report glucagon levels were not reduced by two-months of PAS therapy, thus leading to an imbalance of the insulin:glucagon ratio due to the marked reduction of insulin secretion. The mechanism of this discrepancy can be explained by differential expression of SSTRs subtypes: the α-cell mainly expresses SSTR2, while the β-cell especially SSTR5, whose affinity for PAS is higher, resulting in stronger suppression. Schmid HA et al. pinpointed the importance of insulin:glucagon balance in DM by showing that the co-administration of PAS and octreotide (first generation somatostatin analogue with higher affinity for SSTR2) in rats did not cause a significant increment in glucose levels [27].

The different action of PAS on the two main regulators of glucose metabolism strengthens the idea that an impaired insulin:glucagon ratio may be the pathophysiological basis of PAS-related diabetes. Therefore, it can be speculated that the restoration of this ratio can be a key therapeutic approach to the disease. This objective can be pursued through either enhancing insulin secretion or reducing glucagon tone. The first approach requires exogenous insulin or secretagogues drugs (such as sulfonylureas), whose administration is burdened by the risk of hypoglycemia. On the contrary, reducing glucagon can be achieved safely through incretin mimetics drugs: both dipeptidyl peptidase 4 inhibitors (DPP-4i) and glucagon like peptide 1 analogues (GLP-1a) proved to reduce glucagon levels in a glucose dependent manner, preserving glucagon response to hypoglycemia [28]. Therefore, although our data did not prove an important incretin deficiency in response to meals, incretin-based therapy can represent an effective treatment in PAS-related diabetes [28,29]. Furthermore, GLP-1 analogues, in contrast to DPP-4i, act on typical features associated with chronic GC excess, such as increased appetite, obesity, increased visceral fat mass, altered secretion of adipocytokines and dyslipidemia [20].

The main limitations of our study are the use of indirect measures of insulin sensitivity instead of hyperinsulinemic euglycemic clamp and the lack of incretin-sensitivity measurement. However, MMTT evaluates insulin and incretin secretion immediately, and represents glucose and insulin dynamics of physiological conditions more closely than glucose clamp, mimicking oral challenges routinely encountered daily, which was the aim of the present study. Other limitations are the small number of patients included, especially among those treated with PAS and the lack of control group of non-CD patients with similar metabolic features to determine the extent of GC contribution to DM development. Further studies including a comparable group of non-CD patients with type 2 DM can shed light on the additional effect of GC on DM pathogenesis. Despite all limitations, the major strength of the study is being the first study evaluating incretin secretion in CD during meal consumption and the impact of chronic PAS administration on glucose parameters’ fluctuation. These experimental conditions are close to daily life and give a picture of hormone trend during PAS therapy. In conclusion, insulin resistance is a key determinant of DM in CD and it is related to classic risk factor such as obesity, familiar history and visceral adiposity rather than circulating cortisol levels. A blunted GIP response to MMTT in the DM+ group might also suggest a direct role of hypercortisolism on enteropancreatic axis. PAS treatment confirmed its inhibitory action on insulin secretion but a concomitant improvement in insulin sensitivity due to cortisol reduction was observed as well. Differently from healthy volunteers, no differences in incretin levels and their response to MMTT were recorded in CD patients. The more pronounced reduction in insulin than glucagon levels observed while on PAS, may be an important pathophysiological mechanism in this iatrogenic diabetes; hence restoring insulin:glucagon ratio by either enhancing insulin secretion or reducing glucagon tone can be a potential therapeutic target in this specific setting.

## Figures and Tables

**Table 1:** Anthropometric features and baseline hormone profile of CD patients. SBP, systolic blood pressure; DBP, diastolic blood pressure; F, serum cortisol; UFC, urinary free cortisol; ULN, upper normal limit; SL, salivary.

	DM− (*n* = 19)	DM+ (*n* = 7)	*p*
Age (years)	45 (35.5–55)	54 (41.5–58)	0.37
BMI (kg/m^2^)	24.97 (22.6–26.95)	28.96 (26.6–38.6)	0.012
Waist (cm)	91 (85.75–101)	105.5 (104.25–131.5)	0.006
Hip (cm)	99 (96.25–99.75)	105 (102–119.5)	0.14
SBP (mmHg)	130 (130–137.5)	135 (130–140)	0.47
DBP (mmHg)	90 (80–95)	90 (90–97.5)	0.13
ACTH (ng/L)	39.5 (30.5–62.75)	73 (52–95.5)	0.04
F h 8 (nmol/L)	508 (344.5–579.5)	550.5 (395–628.75)	0.48
UFC/UNL	3.24 (1.62–4.87)	2.56 (1.48–3.5)	0.49
**SL-cortisol h8 (nmol/L)**	12.6 (11.1–14.1)	13.9 (10.2–22.8)	1
**SL-cortisol h23 (nmol/L)**	11.9 (5.15–19.25)	10.2 (7.4–27.55)	0.533
Glycemia (nmol/L)	4.61 (4.5–4.95)	5,8 (4.65–7.55)	0.122
HbA1c (mmol/mol)	35.5 (31.25–36.75)	42 (39–45.5)	0.009
Insulin (mIU/L)	8.5 (6–12.5)	20 (8–29)	0.052
C-peptide (pmol/L)	778 (612.5–999.3)	1589 (1092.6–2016.4)	0.004
GIP (pmol/L)	12.83 (9.53–19.9)	23.23 (13.7–79)	0.078
GLP-1 (pmol/L)	6.53 (5.3–9.96)	8.1 (5.8–14.9)	0.193
Glucagon (pmol/L)	8.53 (3.86–12.75)	9.25 (9.16–14.7)	0.214
Total cholesterol (mg/dL)	188.16 (171–206.75)	215.09 (199.7–257.1)	0.108
HDL(mg/dL)	63 (55.5–69.5)	65.34 (48–103.8)	0.841
LDL (mg/dL)	104.2 (86.5–126)	133.66 (109–160.75)	0.424
Triglyceride (mg/dL)	74.4 (54.5–104.5)	127.88 (96.9–165.8)	0.072
VAI	1.03 (0.67–1.25)	1.31 (0.85–1.89)	0.612
HOMA-B	1.7 (1.1–2.34)	2.45 (1–3.47)	0.545
HOMA-IR	1.81 (1.3–2.45)	3.73 (2.1–8.6)	0.027
HOMA-S (%)	55.35 (40.8–77.2)	26.79 (11.8–47.6)	0.027
FIRI	1.63 (1.17–2.2)	3.36 (1.9–7.7)	0.027
QUICKI	0.35 (0.33–0.37)	0.315 (0.28–0.34)	0.017
Insulin:glucagon ratio	13.63 (9.14–22.82)	10.14 (3.58–26.85)	0.33

**Table 2:** Glycemia, insulin, C-peptide, GIP, GLP-1, and glucagon response to the meal expressed through peak to baseline difference (∆) and net AUC (nAUC) at baseline.

		DM− (*n* = 19)	DM+ (*n* = 7)	*p*
Glycemia	∆ (nmol/L)	3.27 (1.9–4.1)	3.2 (3–5.7)	0.485
	nAUC	3.63 (1.99–5.19)	4.75 (2.875–9.2)	0.115
Insulin	∆ (mIU/L)	107.5 (77.75–163.4)	108 (18–167)	0.586
	nAUC	129.3 (9.36–224.76)	109.75 (20.5–236)	0.809
C-peptide	∆ (pmol/L)	3145.4 (2165.4–3486.4)	2781 (872.4–4006.2)	0.931
	nAUC	4429.2 (3004.7–5619.5)	3757.9 (888.6–5512.7)	0.285
GIP	∆ (pmol/L)	116.5 (84–149.8)	60.6 (35.7–100.85)	0.019
	nAUC	148.71 (106.5–221)	89.97 (55.36–134.35)	0.035
GLP-1	∆ (pmol/L)	8.43 (4.9–13.6)	8.63 (3.64–13.6)	0.623
	nAUC	9.49 (3.57–19.5)	7.19 (2.57–19.1)	0.583
Glucagon	∆ (pmol/L)	6.84 (2.7–11.2)	8.13 (3.22–25.64)	0.544
	nAUC	6.1 (1.14–10.36)	7.16 (2.66–23.34)	0.402

**Table 3:** Anthropometric features and hormone levels in CD patients at baseline and after two months of PAS treatment.

	Baseline	2 Months	*p*
BMI (kg/m^2^)	24.97 (21.85–28.95)	25.01 (23–33.3)	0.203
Waist circumference (cm)	91 (80.5–109.5)	87 (82–104)	0.344
SBP (mmHg)	130 (125–135)	130 (120–136.25)	0.729
DBP (mmHg)	87.5 (77.5–100)	82.5 (80–91.25)	0.493
ACTH (ng/L)	41.9 (31–75)	33.75 (21–73.7)	0.139
S-cortisol h 8 (nmol/L)	510 (447.8–665.8)	514.5 (370.3–566)	0.445
UFC/UNL	3.26 (1.73–3.88)	0.93 (0.4–1.98)	0.005
**SL-cortisol h23 (nmol/L)**	8.3 (3.7–15.3)	4.23 (3.8–5.9)	0.091
Glycemia (nmol/L)	4.8 (4.5–5.1)	5.26 (5–7.6)	0.009
HbA1c (mmol/mol)	35 (29.5–36.5)	41 (36.75–63.25)	0.012
Insulin (mIU/L)	12.9 (7.5–17.35)	4.45 (2–9)	0.008
C-peptide (pmol/L)	857.5 (687–1068.6)	544.65 (278.1–753.2)	0.037
GIP (pmol/L)	14.38 (9.44–25.2)	11.96 (6.8–19)	0.285
GLP-1 (pmol/L)	8.17 (6.5–12.5)	6.47 (4–13.15)	0.646
Glucagon (pmol/L)	9.39 (4.36–15.5)	9.75 (5.4–13.5)	0.646
Total cholesterol (mmol/L)	5.1 (4–5.9)	5.14 (4.28–5.85)	0.499
HDL (mmol/L)	1.71 (1.64–2)	1.6 (1.38–1.76)	0.400
LDL (mmol/L)	2.89 (2.5–4.2)	3.24 (2.33–4.38)	0.735
Triglycerides (mmol/L)	0.67 (0.5–1.14)	0.87 (0.51–2.15)	0.779
VAI	1.06 (0.6–2.2)	0.85 (0.52–2.15)	0.249
HOMA-B	1.89 (1.15–2.69)	0.44 (0.2–0.86)	0.005
HOMA-IR	2.66 (1.5–3.9)	1.1 (0.6–2)	0.007
HOMA-S (%)	37.7 (26.1–66.7)	94.9 (49.8–168)	0.009
FIRI	2.4 (1.37–3.47)	0.99 (0.54–1.83)	0.007
QUICKI	0.33 (0.31–0.36)	0.73 (0.6–0.89)	0.005
Insulin:glucagon ratio	10.3 (7.89–22.2)	5.23 (3.53–7.06)	0.009

**Table 4:** Glycemia, insulin, C-peptide, GIP; GLP-1 and glucagon response to MMTT expressed as peak to baseline difference (∆) and net AUC (nAUC) before therapy and after two months of treatment with PAS.

		Baseline	2 Months	*p*
Glycemia	∆ (nmol/L)	3.1 (2.2–3.83)	3.15 (2.4–4.25)	0.475
	nAUC	3.63 (1.81–5.19)	3.44 (2.2–5.34)	0.799
Insulin	∆ (mIU/L)	109 (80.75–150.75)	40.5 (26–75.75)	0.005
	nAUC	112.46 (97.9–224.8)	53.88 (34.6–82.75)	0.009
C-peptide	∆ (pmol/L)	3195 (2606.5–3395.4)	1930.3 (1519.7–2677.3)	0.017
	nAUC	5763.1 (4186.7–7005)	3082.1 (2536.8–4597.3)	0.022
GIP	∆ (pmol/L)	126.1 (91–228.2)	113.16 (77.5–223.1)	0.241
	nAUC	187.7 (175.9–457.8)	205.7 (141.1–320)	0.203
GLP-1	∆ (pmol/L)	9.29 (4.7–14.1)	10.2 (4.6–12.4)	0.646
	nAUC	8.38 (4.7–26.2)	15.55 (6.52–18.37)	0.799
Glucagon	∆ (pmol/L)	7.91 (1–12.6)	7.1 (4.1–12)	0.799
	nAUC	10.69 (−3–17.14)	8.76 (4.8–14.2)	0.386
